# A comparison between low-dose and standard-dose non-contrasted multidetector CT scanning of the paranasal sinuses

**DOI:** 10.2349/biij.5.3.e13

**Published:** 2009-07-01

**Authors:** SY Lam, SI Bux, G Kumar, KH Ng, AF Hussain

**Affiliations:** 1 Department of Biomedical Imaging, University of Malaya, Kuala Lumpur, Malaysia

**Keywords:** CT Paranasal sinuses, Low-dose Protocol, Chronic sinusitis

## Abstract

**Purpose:**

To compare the image quality of the low-dose to the standard-dose protocol of MDCT scanning of the paranasal sinuses, based on subjective assessment and determine the radiation doses to the eyes and thyroid gland and dose reduction between these two protocols.

**Materials and Methods:**

31 adult patients were scanned. Prior to scanning, thermoluminescent dosimeters (TLDs) were placed at 4 sites: outer canthus of right eye, outer canthus of left eye, inner canthus and anterior neck (thyroid gland). Every patient was scanned twice using the standard-dose protocol (100mAs) followed by the low-dose protocol (40mAs). The images were reviewed by 3 radiologists. Wilcoxon test was used as the test of significance for the image quality assessments. The paired sample t-test was used as the test of significance for the analysis of the radiation doses measured by the TLDs.

**Results:**

Of the 30 patients selected for analysis, this study showed no significant difference in the scores for the diagnostic image quality and the anatomical structures assessments between the two protocols. The average calculated mean entrance surface doses and standard deviation for the standard-dose and low-dose protocols were 12.40±1.39 mGy and 5.53±0.82 mGy respectively to the lens and 1.03±0.55 mGy and 0.63±0.53 mGy respectively to the thyroid gland.

**Conclusion:**

The reduction of mAs from 100 to 40 resulted in a significant reduction of the radiation doses to the lens and thyroid gland by 55.4% and 38.8% respectively without causing any significant effect to the diagnostic image quality and assessment of the anatomical structures.

## INTRODUCTION

Sinusitis is one of the most common health care problems worldwide and there is evidence that it is increasing in prevalence and incidence. In patients suspected to have acute sinusitis, this stage is usually treated medically and radiological investigation is rarely required.

While plain radiographs have often been used as part of the initial workup of patients with suspected chronic sinusitis, it is well known that the sensitivity of plain radiography in diagnosing this condition is much lower than computed tomography (CT) as interpretation is fraught with difficulty due to the great variation in normal appearance of the paranasal sinuses and the presence of many complex overlapping structures. Plain radiographs also have low specificity and sensitivity when compared with clinical and surgical findings [1]. In 1995, the Royal College of Radiologists Working Party [2] said that plain radiographs have no place in the routine management of rhinosinusitis.

Thus, CT has become the method of choice for confirming and determining the extent of the disease. In addition, with the progress of effective surgical techniques for chronic sinusitis such as functional endoscopic sinus surgery (FESS), which has been increasingly employed in the treatment of sinus disease, high-quality CT has become a well-established mandatory preoperative diagnostic tool. This is due to the advantage of CT in providing detailed information of the highly variable anatomy of the nasal cavities and paranasal sinuses as well as the relationship of the diseased areas to vital structures such as the optic nerve and internal carotid artery, thereby providing a ‘roadmap’ for endoscopic surgery [3-5].

However, the known disadvantage of CT imaging is the radiation exposure and the most radiosensitive organs within the scanning field are the thyroid gland and the eye lens, in which the latter organ is at risk for radiation induced cataract [6-7]. Thus, limiting and reducing radiation dose to the eye is important, especially in young patients and in patients who require repeated scanning in which they are subjected to cumulative radiation exposure of multiple scans.

The tube current setting during scan acquisition is the most important parameter that affects radiation dose and image quality. Ideally, the tube current setting is selected at those that use the minimum radiation required for diagnostic image quality. From the early nineties, in view of the high inherent contrast structures in sinus CT, attempts have been made to adopt low-dose methods as the diagnostic quality of images of these high inherent contrast structures (normally viewed with window width of +2000) is not substantially affected by a worsening of signal-to-noise ratio even when the scanning is done in very thin slices [8-15]. With the advent of multi-detector CT (MDCT) scanners with excellent multiplanar reconstruction, excellent image quality can be produced at a much lower radiation dose.

## OBJECTIVES

This paper’s general objective is to determine if the image quality of the low-dose protocol MDCT scanning of the paranasal sinuses has any significant difference to the image quality of standard-dose protocol based on subjective assessment, for the diagnosis and management of patient with chronic sinusitis. Specifically, we compare the diagnostic image quality and the delineation of the important and clinically relevant anatomical details of the nasal cavities and paranasal sinuses between low-dose and standard-dose MDCT scanning of the paranasal sinuses. The absorbed eye and thyroid doses of both protocols were also measured.

## METHODOLOGY

### Patient selection and Period of Study

This prospective study was carried out on 31 adult patients referred to the Biomedical Imaging Department of University Malaya Medical Centre (UMMC) for CT of the paranasal sinuses. The period of data collection was from October 2005 till October 2006. This study was approved by the Medical Ethics Committee, UMMC (MEC. Ref. No. 465.15) and supported by University Malaya (UM), Vote F (F0217/2005C), Short-Term Research Fund.

### Inclusion and exclusion criteria

The patients who were scheduled for non-contrasted CT scanning of the paranasal sinuses were those suspected to have chronic sinusitis or recurrent chronic sinusitis or nasal polyp. Patients who had been selected for preoperative assessment for FESS and for further assessment of the paranasal sinuses (post-FESS) were also included in this study. The patients who were less than 18 years old or suspected to have other paranasal sinus pathology such as tumours or fractures were excluded from this study.

### Procedure and Methods

#### Pre-scanning procedure

Informed consent was obtained from all patients in the study and any radio-opaque objects were removed from the patients’ head and neck region. Patients’ height, weight, anteroposterior and biparietal head measurements were carried out. The patients were scanned in the supine position. After obtaining the topogram and prior to the first scanning, two lithium fluoride thermoluminescent dosimeter (TLD) chips aligned in a plastic sachet were attached to the patient’s skin parallel to the beam slice using surgical plaster on each of the following 4 sites: outer canthus of right eye, outer canthus of left eye, inner canthus and anterior neck (thyroid gland).

#### Scanning Protocols and Reconstruction

Non-contrasted helical scanning was performed using the 16-slice CT scanner (Siemens SOMATOM Sensation 16, Forschheim, Germany) in axial sections covering the region from the top of the frontal sinuses to the hard palate and from the tip of the nose to the region just posterior to the mastoid air cells. The scans were acquired in a cranio-caudal order.

Each of the patients was scanned twice, first using the standard protocol followed by the low-dose protocol. Prior to the second scanning, the TLDs were removed and replaced by another new set of TLDs attached on the same sites. Both protocols comprised a fixed KVp of 120, slice collimation of 16 x 0.75 mm, slice width of 3.0 mm, feed per rotation of 6.0 mm, rotation time of 0.5 s and pitch of 0.55. The only parameter that was varied in this study is the effective mAs, which was 100 in the standard-dose protocol and 40 in the low-dose protocol.

Both the scans were acquired using Kernel H60f sharp and in osteo window. A 512 x 512 image matrix was used for both scans. From the volumetric raw data of both scanning protocols, the images of each patient were then reconstructed in 1.0 mm with an increment of 0.8 mm and saved in a compact disc.

### Data Analysis

#### Image Quality

Prior to data analysis, the images in the compact disc of each patient were loaded into the GE Advantage Workstation AW 4.2_07. The reformatted axial and coronal images of the standard-dose and low-dose protocols of each patient were then independently evaluated by three experienced radiologists at different times. The readers were blinded to the mAs setting used and the images were viewed in bone window setting (window width of 2000 and window level of 350) and in the same viewing condition. The images were reviewed and scored only once by the three radiologists.

The image quality was assessed based on the following criteria. First, the diagnostic image quality was assessed by the complete opacification of one or more of the sinuses, presence of mucosal thickening, air-fluid level, any bony abnormalities (sclerosis, thickening or lysis), deviation of nasal septum and turbinate hypertrophy. Scores were ascribed as follows: 0 if the radiological finding is not seen, 1 if the radiological finding is visible but indeterminate and 2 if the radiological finding is clearly seen. Secondly, the following important and clinically relevant anatomical structures of the nasal cavities and paranasal sinuses were assessed: the maxillary sinuses, osteomeatal complex (including the ethmoidal infundibulum, uncinate process, maxillary ostium, ostia of anterior and middle ethmoidal air cells and middle meatus), frontal sinus, frontal recesses, anterior ethmoidal air cells (including the agger nasi cells-frontal anterior ethmoidal air cells), posterior ethmoidal air cells, basal lamina (divides the anterior and posterior ethmoidal air cells), sphenoethmoidal recess (including the ostium of the sphenoid sinus), sphenoid sinus and septum, cribriform plate, lamina papyracea, the path of both optic nerves, including its relation to the posterior ethmoidal air cells and both internal carotid arteries (ICA) in relation to the sphenoid sinus. The reviewers were asked to judge whether the appearance of the anatomic structures was normal, indeterminate or abnormal. Scores were again ascribed as follows: 0 if the structure is normal, 1 if the structure is indeterminate and 2 if the structure is abnormal. Mucosa was considered to be normal if it was not visible and was considered abnormal (thickened) if it was visible. Indeterminate findings included those instances in which a reviewer was doubtful or in which the anatomic structure was not seen (e.g., the osteomeatal unit after a previous surgery of the maxillary sinus or anatomical variations such as non-pneumatization of the sinuses). The bones were considered abnormal if there were any sclerosis, thickening or lysis noted. The frontal and sphenoethmoidal recesses were considered abnormal if the recesses were not seen to be patent.

For each scan, scores for the diagnostic image quality were then added together to achieve an overall quality rating. Thus, the minimum possible score for diagnostic image quality assessment was 0 and maximum score was 12. Similarly, for each scan, scores for the important and clinically relevant anatomical structures of the nasal cavities and paranasal sinuses assessment were added together to achieve an overall quality rating and the minimum possible score for this assessment was 0 and the maximum score was 30.

#### Inter-observer Variability

The coefficient of variance was calculated from the total scores of the diagnostic image quality and anatomical structures assessment for the standard-dose and low-dose protocols respectively. The overall interobserver variability and the inter-observer variability between the respective reviewers were determined.

#### Radiation Dose Measurement

The TLDs of LiF:Mg,Cu,P (Harshaw TLD-100H) were used for the dose measurement. An ionization chamber (Model Radcal 10X5-60) was used for calibration of the TLDs. This ionization chamber system was calibrated annually by the Malaysian Nuclear Agency which is the Secondary Standard Dosimetry Laboratory. For the sensitivity test, 100 chips were exposed at air kerma of 100 mR. Only the chips that have sensitivity within 10% of the mean value were used for this study. The linearity of these TLDs was tested over the range of 150 mR to 750 mR and the results showed a good linear fit with a coefficient of determination (R^2^) of 0.997. The calculated response of the TLDs from tube voltage 120 kV was taken as the calibration factor in this study. Prior to use, all chips were annealed for 10 minutes at 240°C in a nickel-plated copper annealing stack. The TLD readings of each patient were carried out after the scanning and calculations of the equivalent dose to the eyes and thyroid gland were done. TLD dose of the eye was assumed to be equal to the dose delivered to the lens. The respective estimated effective doses (ED) and organ doses for male and female of both scanning protocols were also obtained from commercially available software i.e. WinDose 2.1a, Institute of Medical Physic, Erlangen, Germany, and were compared to the calculated dose from the TLD. Two methods of quantifying dose in CT are the dose-length product (DLP) measured in mGy.cm and the weighted CT dose index (CTDIw).

Data was analyzed using the software Microsoft Excel 2000 and SPSS version 12.0 for Windows. The mean total scores from the low-dose protocol were compared to the mean total scores from the standard dose protocol of each reviewer for both diagnostic image quality and relevant anatomical structures assessments. The null hypothesis was that there is no difference between these scores. The non-parametric Wilcoxon signed rank test was used as the test of significance and the p-value of less than 0.05 was considered statistically significant. The parametric paired sample t-test was used as the test of significance for the comparison between the radiation doses (entrance surface dose) measured by the TLD during CT scanning of the paranasal sinuses using standard-dose and low-dose protocol, respectively. The calculations were done at 95% confidence interval.

## RESULTS

### Basic demographic data and characteristics of studied patients

Of the 30 patients selected for the analysis of this study, 17 (56.7%) were male and 13 (43.3%) were female. One patient was excluded from the analysis of this study due to incomplete CT image acquisition and inaccurate radiation dose measurement. The patients’ ages varied from 20 to 72 years old with a mean age of 43.5 years. Their heights and weights ranged from 140.0 cm to 176.0 cm and from 39.0 kg to 92.8 kg, respectively, with mean height of 160.8 cm and mean weight of 62.3 kg. The head measurement in anteroposterior (AP) and biparietal (BP) diameters of the patients varied from 17.3 cm to 20.0 cm and from 13.0 cm to 16.8 cm with mean of 18.5 cm and 15.2 cm, respectively (Table 1).

**Table 1:** Patient Characteristics.

		**Mean ± SD**	**Range**
Age (years)		43.5 ± 15.9	20 - 72
Height (cm)		160.8 ± 9.1	140 - 176
Weight (kg)		62.3 ± 13.6	39.0 - 92.8
Head measurement (cm)	AP diameter	18.5 ± 0.68	17.3 - 20.0
	BP diameter	15.2 ± 0.93	13.0 - 16.8

N = 30

### Clinical data of studied patients

The indications for the CT scanning in these studied patients were acute and chronic sinusitis, frontal headache, nasal polyp and for pre-operative assessment prior to FESS. Three of the patients included in this study had history of previous sinus surgery. One of the patients who had previous nasopharyngeal carcinoma 20 years ago, was treated with radiotherapy and in remission but subsequently developed chronic sinusitis was also included in this study. This was thought not to affect the general and specific objectives of the study which was to compare the diagnostic image quality of both protocols.

### Diagnostic image quality assessment

Based on the analysis of the overall total score of both protocols by the first reviewer (radiologist 1), there were differences seen in the scores of five studied patients. However, these differences have been proven to be statistically not significant (Table 2). As for the second and third reviewers (radiologist 2 and 3), there was no difference in the total scores of all the CT images reviewed.

**Table 2:** Mean scores and significance (p-value) of the overall diagnostic image quality assessment of the CT images at both protocols (based on Wilcoxon's Signed Rank Test).

**Reviewers**	**Mean Total Score**		**Significance**
	**Standard-dose (100 mAs)**	**Low-dose (40 mAs)**	**p value**
Radiologist 1	6.17	6.20	p = 0.89 (NS)
Radiologist 2	6.27	6.27	p = 1.0 (NS)
Radiologist 3	4.93	4.93	p = 1.0 (NS)

N = 30

In the analysis of the actual score of the individual radiological findings, there were differences seen between the two protocols of five patients on the assessment of the bony abnormalities, deviation of nasal septum and turbinate hypertrophy, especially the bony abnormalities i.e. bony lysis on three of the studied patients by the first reviewer. However, these differences again have been proven to be statistically not significant (Table 3). Similarly, there was no difference in the actual score of all the individual radiological findings by the second and third reviewers.

**Table 3:** Comparison between the standard-dose and low-dose protocols of the selected radiological findings (based on Wilcoxon's Signed Rank Test).

**First Reviewer (Radiologist 1)**	**Positive Rank**	**Tie**	**Negative Rank**	**p value**
Bony abnormalities	2	27	1	p = 0.78 (NS)
Nasal Septum Deviation	0	29	1	p = 0.32 (NS)
Turbinate Hypertrophy	1	29	0	p = 0.32 (NS)

N = 30

### Assessment of anatomical structures

Based on the analysis of the overall total score of both protocols by the first reviewer (radiologist 1), there were differences seen in the scores of seven studied patients. However, these differences have been proven to be statistically not significant (Table 4). As for the second and third reviewers (radiologist 2 and 3), there was no difference in the total scores of all the CT images reviewed.

**Table 4:** Mean scores and significance (p-value) of the overall assessment of the selected anatomical structures on CT images at both protocols (based on Wilcoxon's Signed Ranks Test).

**Reviewers**	**Mean Total Score**		**Significance**
	**Standard-dose (100 mAs)**	**Low-dose (40 mAs)**	**p value**
Radiologist 1	20.0	20.0	p = 0.89 (NS)
Radiologist 2	18.8	18.8	p = 1.0 (NS)
Radiologist 3	15.7	15.7	p = 1.0 (NS)

N = 30

In the analysis of the actual score of the individual anatomical structures, there were differences seen between the two protocols of seven patients on the assessment of the frontal recesses, basal lamina and sphenoethmoidal recess, especially the frontal recesses on five of the studied patients by the first reviewer. However, these differences again have been proven to be statistically not significant (Table 5). Similarly, there was no difference in the actual score of all the individual radiological findings by the second and third reviewers.

**Table 5:** Comparison between the standard-dose and low-dose protocols of the selected anatomical structures (based on Wilcoxon's Signed Ranks Test).

**First Reviewer (Radiologist 1)**	**Positive Rank**	**Tie**	**Negative Rank**	**p value**
Frontal recesses (Right/Left)	2/3	25/25	3/2	p = 0.48 / p = 1.0 (NS)
Basal lamina (Right and Left)	2	28	0	p = 0.16 (NS)
Sphenoethmoidal recess (Left)	0	29	1	p = 0.32 (NS)

N = 30

### Inter-observer variability

The overall coefficient of variance (CoV) that was calculated for the diagnostic image quality assessment of the standard-dose and low-dose protocols were 24.8% and 26.2%, respectively. The CoV that was calculated for this assessment between the first and second reviewers for both protocols were 4.1% and 5.7%, respectively. The calculated CoV between the first and third reviewers for both protocols were 30.7% and 34.1%, respectively, and between the second and third reviewers were 33.9% for both protocols, respectively (Table 6).

**Table 6:** Inter-observer variability as shown by the coefficient of variance for the total scores of the diagnostic image quality and anatomical structures assessment of standard-dose and low-dose protocols.

	**Coefficient of variance (%)**			
	**Diagnostic image quality assessment**		**Anatomical structures assessment**	
	**Standard-dose protocol**	**Low-dose protocol**	**Standard-dose protocol**	**Low-dose protocol**
Overall	24.8	26.2	25.8	26.0
Between 1st and 2nd reviewers	4.1	5.7	6.1	6.2
Between 1st and 3rd reviewers	30.7	34.1	38.2	38.6
Between 2nd and 3rd reviewers	33.9	33.9	35.8	35.8

The overall CoV that was calculated for the anatomical structure assessment for the standard-dose and low-dose protocols were 25.8% and 26.0%, respectively. The CoV that was calculated for this assessment between the first and second reviewers for both protocols were 6.1% and 6.2%, respectively. The calculated CoV between the first and third reviewers for both protocols were 38.2% and 38.6%, respectively, and between the second and third reviewers were 35.8% for both protocols, respectively (Table 6).

### Radiation dose measurement

Radiation dose measurement using the TLD was only done on 23 patients in this study as the TLDs were not ready for use during scanning of the remaining eight patients. However, the result of one of the patients was excluded as there was a technical error during scanning in which the scanner stopped scanning halfway during the low-dose protocol and the doses measured were unacceptably higher than expected.

The average calculated mean entrance surface doses and standard deviation based on the TLD measurement during CT scanning of the paranasal sinuses using the standard-dose and low-dose protocol were 12.40±1.39 mGy and 5.53±0.82 mGy, respectively, to the lens and 1.03±0.55 mGy and 0.63±0.53 mGy, respectively, to the thyroid gland. The mean dose reduction to the eyes and thyroid gland was 55.4% and 38.8%, respectively. Significant reduction in the mean doses between the two protocols is seen at 95% confidence interval (Table 7). Similarly, the weighted CT dose index (CTDIw) and dose-length product (DLP) showed reduction in the mean dose of 59.6% and 59.7%, respectively, when the mAs was reduced from 100 to 40 (Table 8).

**Table 7:** Radiation Doses (Entrance Surface Dose) based on TLD measurement during CT scanning of the paranasal sinuses using standard-dose and low-dose protocols (based on Paired-sampled T-Test).

**Locations**	**Mean Entrance Surface Dose ± SD (mGy)**		**Mean Dose Reduction (mGy)(%)**	**Significance (p value)**
	**Standard-dose (100 mAs)**	**Low-dose (40 mAs)**		
Outer canthus of right eye	12.54 ± 1.09	5.53 ± 0.68	7.01 (55.9)	p < 0.001
Outer canthus of left eye	12.33 ± 1.62	5.67 ± 0.72	6.66 (54.0)	p < 0.001
Inter canthus	12.33 ± 1.45	5.38 ± 1.05	6.95 (56.4)	p < 0.001
Anterior Neck (Thyroid)	1.03 ± 0.55	0.63 ± 0.53	0.40 (38.8)	p < 0.001

N=22

**Table 8:** CT Methods of Dose Quantification of both protocols.

**CT Methods of Dose Quantification**	**Standard-dose (100 mAs)**	**Low-dose (40 mAs)**	**Mean Dose Reduction (%)**	**Significance (p value)**
CTDIw	21.23	8.57	59.6	-
DLP (mGy.cm)	272.82 ± 26.45	109.91 ± 10.87	59.7	p < 0.001

N=30

## DISCUSSION AND CONCLUSION

The advent of new generation CT scanners with improved spatial and contrast resolution had provided the potential to maintain scan quality at a much lower radiation dose and thus, revolutionized diagnostic imaging. In view of the high inherent contrast structures in sinus CT, attempts have been made to adopt low-dose methods in many studies and had proven not to affect the diagnostic image quality. However, it is not uncommon for an imaging department to adhere to the standard imaging protocol without being aware of radiation dose reduction potentialities.

The tube current setting in the standard protocol in the authors’ department was 100 mAs using the 16-slice CT scanner (Siemens SOMATOM Sensation 16). Tube current setting of 40 mAs was employed in the low-dose protocol based on the recommended parameter from the scanner’s manufacturer and was the lowest mAs possible for scanning. During the pilot study, the CT scanner was unable to scan at the tube current setting below 40 mAs. The assessment of image quality was divided into two parts; the diagnostic and associated radiological findings of acute or chronic sinusitis and its complications, and the clarity of the anatomical structures which are important to the ENT surgeons for pre-operative assessment.

The authors’ study results show that the diagnostic and associated radiological features of acute or chronic sinusitis can be clearly visualized on the low-dose protocol scans with no significant difference to the standard-dose protocol despite some increase in noise (graininess). Individually, there were no discrepancies in the total and individual scores between the two protocols given by the second and third reviewers and only small discrepancies in the scores given by the first reviewer.

As for the first reviewer, the differences in the individual scores of three radiological findings were noted, namely bony abnormalities, deviation of nasal septum and turbinate hypertrophy. The subjective assessment of these structures may differ at different time of reading by a reviewer especially if the abnormality is of a mild degree.

As for the assessment of the clarity of the important anatomical structures, again the authors found that there was no significant difference between the standard-dose and low-dose protocol scans. Individually, there were again no discrepancies in the total and individual scores between the two protocols given by the second and third reviewers and only small discrepancies in the scores given by the first reviewer. In the first reviewer, the differences in the individual scores of three anatomical structures were noted i.e. frontal recesses, basal lamina and sphenoethmoidal recess. Due to anatomical variation in different patients, these structures were not clearly identified in some of the scans causing discrepancies in the assessment of these structures. Thus, it was thought that these discrepancies were not due to the effect of the different protocols used in the scanning.

The overall coefficient of variance (CoV) reflecting the inter-observer variability of the three reviewers in the diagnostic image quality assessment was 28.4% for the standard-dose protocol and 26.2% for the low-dose protocol. However, the CoV calculated between the first and second reviewers were small (4.1% and 5.7%, respectively) in contrast to the values calculated between the first and second reviewers and the third reviewer, respectively (30-34%). Similar results were also seen with the anatomical structures assessment in which the overall CoV calculated was 25.8% for the standard-dose protocol and 26.0% for the low-dose protocol. The CoV calculated between the first and second reviewers were also small (6.1% and 6.2%, respectively) in contrast to the values calculated between the first and second reviewers and the third reviewer, respectively (35-38%). The difference in the experiences of the three reviewers is the most likely cause for the high interobserver variability, particularly with the third reviewer.

However, the authors’ overall findings are agreeable with some of the results of previous studies which suggest that low mAs settings do not adversely affect the diagnostic image quality and bony details in the CT scanning of paranasal sinuses for acute or chronic sinusitis. Duvoisin et al [9] concluded that tube setting of as low as 30 mAs is sufficient for analysis of normal and abnormal structures. Kerney et al [10] found that the overall perceived quality of the scans and clarity of important anatomical structures are not affected by scanning at 40 mAs. Sohaib et al [11] also concluded that important anatomical structures can be clearly seen on scan done at 50 mAs. The results of these three studies are in good agreement with the results of the authors’ study. Recently, a study done by Brem et al [16], using computer simulation of the effect of low-radiation dose acquisition on MDCT of paranasal sinuses, identified an effective tube current of 67 mAs in providing sufficient diagnostic quality for the bone structures studied (nasal septum, middle and inferior turbinates, and frontal sinus). They also found that a higher effective tube current of 134 mAs allows adequate visualization of both soft tissue and bone structures. Attempts to further lower the radiation dose possibly equal or lower than the radiation exposure of four-view radiographic examination had also been done. In the study done by Marmolya et al [8], they concluded that except for defining bone landmarks and borders of the sinuses, scans done at tube current setting as low as 16 mAs on axial scanning are still diagnostic for sinusitis and ostia narrowing can be seen as a cause. Tack et al [13] concluded that the dose reduction as low as 10 mAs played a far less important role in discrepancies of detected abnormalities than did the human element of reviewer observation and suggested that low-dose MDCT scanning of the paranasal sinuses should be considered the imaging method of choice in patients with suspected chronic sinusitis. However, the results of these two studies are not comparable with the authors’ findings. In the sample population of this study, the weight of the patients varied from 39.0 kg to 92.8 kg and the mean antero-posterior and biparietal diameter of the head are 18.5 cm and 15.2 cm with standard deviation of 0.68 cm and 0.93 cm, respectively. Although, there is a large difference in the patient’s weight of 53.8 kg but this variable is less important in the assessment of the paranasal sinus region. Head size is a more important variable that affects the radiation dose measurement, but this study shows that the difference in the head size of adult population is less than 2 cm, which is small, and therefore, will not significantly affect the radiation dose measurement.

In this study, the TLD doses at the outer cantus of both eyes, inter cantus and anterior neck regions are assumed to be equal to the dose delivered to the eye lens and thyroid gland respectively. The result of this study shows that the radiation dose to the eye lens is 12.40±1.39 mGy at 100 mAs and 5.53±0.82 mGy at 40 mAs and the radiation dose to the thyroid gland is 1.03±0.55 mGy at 100 mAs and 0.63±0.53 mGy at 40 mAs. These doses are almost comparable to the organ doses obtained from commercially available software i.e. WinDose 2.1a (Institute of Medical Physics, Erlangen, Germany) in which the eye lens doses are 15.40 mSv for male and 16.01 mSv for female at 100 mAs, and 6.16 mSv for male and 6.40 mSv for female at 40mAs, and the thyroid doses are 0.96 mSv for male and 0.92 mSv for female at 100 mAs and 0.38 mSv for male and 0.37 mSv for female at 40mAs. These results compare favourably with other recent studies done. In the study done by Zammit-Maempel et al in 2003 [7], using Siemens Somatom Volume Zoom quad slice CT scanner, they found that the TLD measurement at the parameters of 120 kV and effective mAs of 100 showed mean lens dose of 28.7 mGy and thyroid dose of 1.3 mGy and at the parameters of 120 kV and effective mAs of 40, the mean lens dose was 9.2 mGy and thyroid dose was 0.4 mGy. The average scan time was 11s with 1 mm collimation used but the pitch was not mentioned in this study. Cathcart et al in 2002 [12] using a Toshiba Express Helical CT scanner with parameters of 120 KVp, 50 mAs and pitch ratio of 1.6, reported the estimated radiation dose to eye lens to be 5 mGy. Sohaib et al in 2001 [11] reported that the mean absorbed dose to the lens ranges from 2.0- 14.3 mGy at 100 mAs to 1.0-5.6 mGy at 50 mAs using GE HiSpeed Advantage CT scanner with other parameters of 120 KVp and 1 s per slice at 5 mm table increments of sequential scanning.

In other words, this study shows that there is a significant reduction of the doses to the lens of the eye and thyroid gland by 55.4% and 38.8%, respectively when the mAs is reduced from 100 to 40. Although the eye lens dose measured during CT scanning of the paranasal sinuses at standard dose protocol is substantially less than the cumulative radiation exposure of 0.5-2 Gy believed to induce corneal opacities [17] and the radiation exposure of over 5 Gy to cause visual impairment due to cataract, this dose reduction is still important. There is a theoretical risk of non-deterministic effects in which the radiation exposure is cumulative in nature in its effect on the eye lens particularly to some patients requiring multiple imaging.

The calculated effective doses according to Publication 60 of the International Commission of Radiation Protection (ICRP) using the software WinDose 2.1a (Institute of Medical Physic, Erlangen, Germany) at 100 mAs are 0.68 mSv for male and 0.66 mSv for female, and at 40 mAs are 0.27 mSv for male and 0.26 mSv for female. This study further shows that by using a lower mAs with an effective dose of approximately 0.3 mSv, the risk of fatal cancer is estimated at 1 in 67,000 as compared to 1 in 30,000 for an effective dose of approximately 0.7 mSv if the standard mA is used [18]. There is also evidence of dose reduction of 59.6% and 59.7% based on the CTDIw and DLP values, respectively, when a lower mA is used. In addition, reducing the mA setting also has the advantage of reducing tube loading and prolonging the life of the X-ray tube [12]. Based on a current review article on CT as an increasing source of radiation exposure [19], there has been a great concern on the increasing radiation exposure in a given population and the main concern is on radiation-induced carcinogenesis. A recent large-scale study of 400,000 radiation workers in the nuclear industry showed a significant increase in the risk of cancer among these workers who received doses between 5 and 150 mSv. Similar findings were also reported in the subgroup of atomic-bomb Japanese survivors. Although the individual risk estimates are small, when applied to an increasingly large population may lead to a public health issue in the future. There had been a rapid increase in the use of CT scans in many countries, notably in Japan. According to a survey conducted in 1996, the number of CT scanners per 1 million population was 64 in Japan and 24 in United States. An exponential increase in the number of CT scans performed annually in the United States had also been reported. It is estimated that more than 62 million CT scans are currently obtained each year in United States, as compared to about 3 million scans in 1980 [19]. In Malaysia, there was also a notable increase in the use of CT by 161% from 1990 till 1994, based on a national survey [20, 21], which is the only latest data available and reported.

One of the ways to reduce radiation dose from CT in the population is to reduce the CT-related dose in individual patients. Thus, reduction of CT dose should be attempted wherever is possible, as shown and proven in this study. In addition, children are at greater risk than adults in developing radiation-induced cancers as they are inherently more radiosensitive. A low-dose protocol for CT scanning of the paranasal sinuses in paediatric patients should also be employed. There are some limitations in this study. The first limitation is that the assessments were done on a noncontrasted CT scan of the paranasal sinuses. Thus, the results of this study will not be applicable to the contrasted CT scanning of the paranasal sinuses for other indications such as sinus tumours or abscesses. Secondly, the low-dose protocol used i.e. 40 mAs may not be directly transferable to different scanner types as the lowest mAs value which will not affect the diagnostic image quality of the examination may be higher. Based on this study, the lowest mAs sufficient for diagnostic quality is 40. Further reduction of the mAs is thought to be possible without compromising diagnostic quality. Further studies using lower mAs can be done to further reduce the radiation dose to the patient. The radiation dose measurements were done on the actual CT scanning, excluding the topogram. Hence, the actual radiation dose to the patient should be more than the calculated value. The radiation dose measured in this study may be different with other scanners of different manufacturer even with the same tube current setting.

In conclusion, despite the limitations in this study, the authors strongly recommend the employment of low dose technique in all non-contrasted CT scanning of the paranasal sinuses.

**Figure 1:**
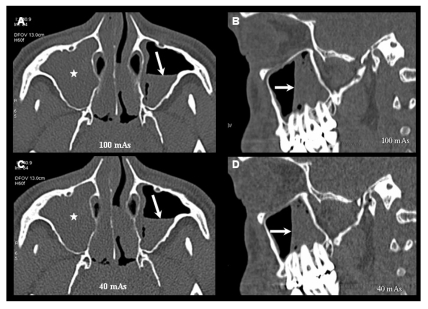
Axial and Sagittal images of MDCT scans obtained at 100 mAs (A, B) and 40 mAs (C, D) at the level of the maxillary sinuses show complete opacification of the right maxillary sinus (star) and air-fluid level in the left maxillary sinus (arrow). No significant difference in the diagnostic image quality of these two radiological findings of these two scans.

**Figure 2:**
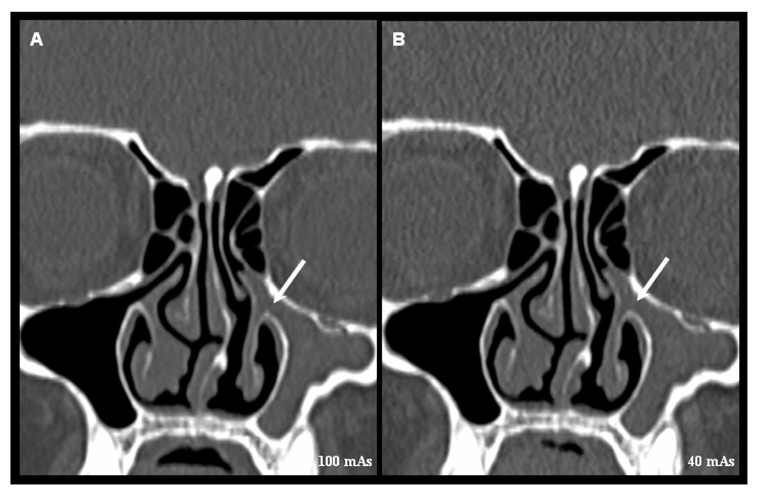
Coronal reformatted images of MDCT scans obtained at 100 mAs (A) and 40 mAs (B) at the level of osteomeatal complex showing a normal right osteomeatal complex and a blocked left osteomeatal complex (arrow). This structure is clearly identified and correctly assessed in these two scans of this patient.

**Figure 3:**
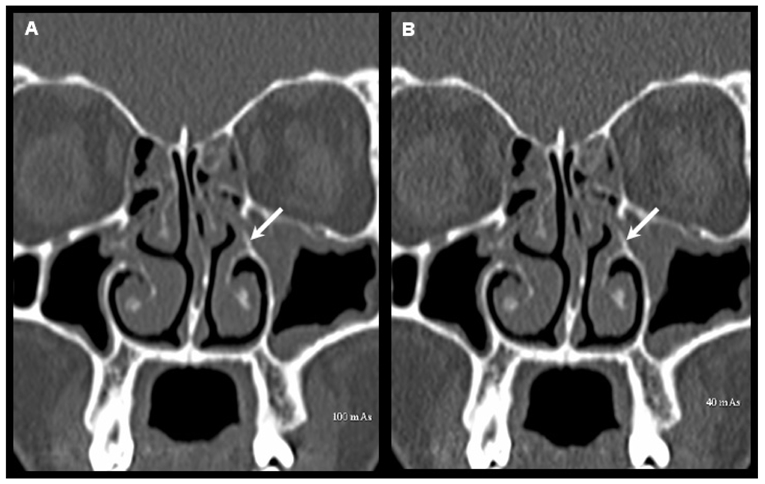
Coronal reformatted images of MDCT scans obtained at 100 mAs (A) and 40 mAs (B) at the level of osteomeatal complex showing the left uncinate process (arrow). This structure is clearly identified and correctly assessed in these two scans of this patient.

**Figure 4:**
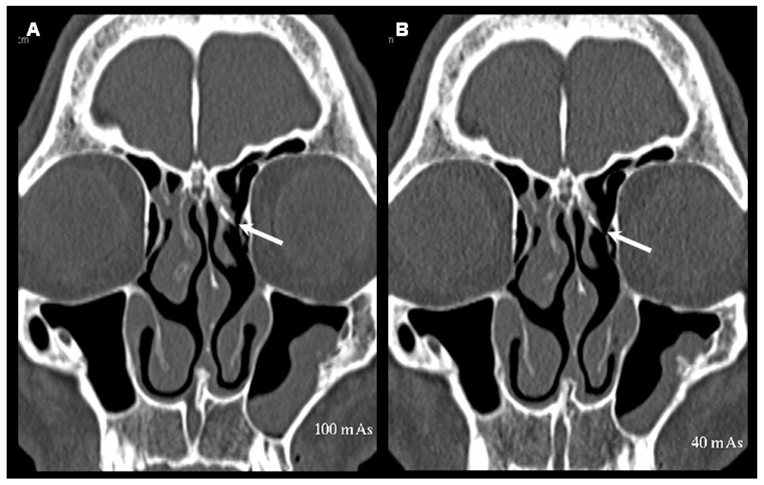
Coronal reformatted images of MDCT scans obtained at 100 mAs (A) and 40 mAs (B) at the level of frontal recess showing a normal left frontal recess (arrow) and a blocked right frontal recess. This structure is also clearly identified and correctly assessed in these two scans of this patient.

**Figure 5:**
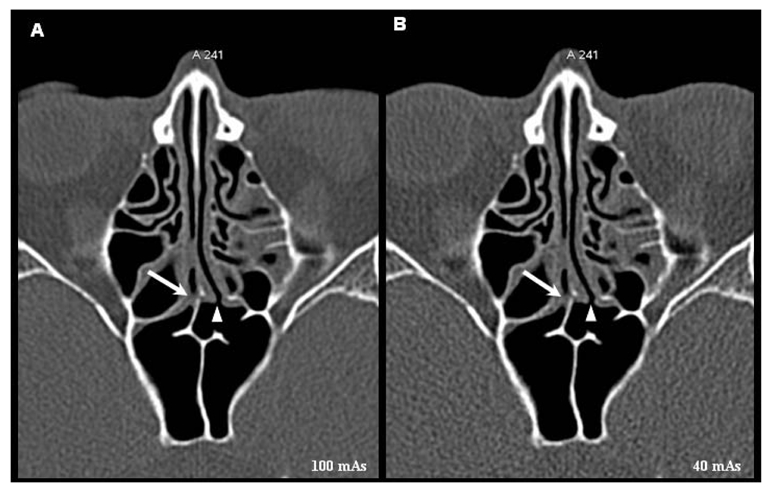
Axial images of MDCT scans obtained at 100 mAs (A) and 40 mAs (B) at the level of the ethmoidal sinuses showing an abnormal (blocked) right sphenoethmoidal recess (arrow) and a normal (patent) left sphenoethmoidal recess (arrow head) which are clearly seen in these two scans.

**Figure 6:**
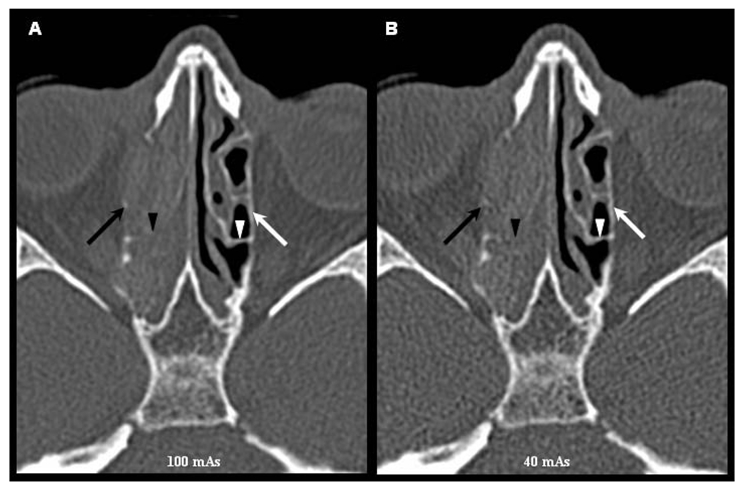
Axial images of MDCT scans obtained at 100 mAs (A) and 40 mAs (B) at the level of the ethmoidal sinuses showing erosion of the right lamina papyracea (black arrow) and basal lamina (black arrow head). The left lamina papyracea (white arrow) and left basal lamina (white arrow head) are preserved. These structures are clearly seen in these two scans.
